# Biomimetic co-delivery nanoplatform overcomes CAF- and TAM-induced immunosuppression to augment therapeutic efficacy against triple-negative breast cancer

**DOI:** 10.1016/j.ijpx.2026.100597

**Published:** 2026-07-02

**Authors:** Haiwang Luo, Di Cao, Mei Ma, Xinyi Lu, Jing Wang

**Affiliations:** a School of Pharmacy, Ningxia Medical University, 1160 Shengli Street, 750004 Yinchuan, China; b Key Laboratory of Protection, Development and Utilization of Medicinal Resources in Liupanshan Area (NXMU), Ministry of Education, 692 Shengli Street, 750001 Yinchuan, China

**Keywords:** Immunotherapy, TNBC, Biomimetic nanomedicines, CAFs, TAMs, Hedgehog pathway

## Abstract

Immune checkpoint blockade (ICB) has emerged as a cornerstone therapy for triple-negative breast cancer (TNBC); however, its clinical efficacy remains suboptimal due to the immunosuppressive tumor microenvironment (TME). TNBC is characterized by persistent activation of cancer-associated fibroblasts (CAFs) and infiltration of immunosuppressive cells, particularly tumor-associated macrophages (TAMs). Notably, the Hedgehog (Hh) signaling pathway plays a crucial role in both CAF activation and TAM polarization. To address these limitations, we developed an erythrocyte membrane-camouflaged biomimetic co-delivery nanoplatform encapsulating Vismodegib and BMS-1 (designated as E-V/B@NM). *In vitro* and *in vivo* studies demonstrated that the fabricated E-V/B@NM significantly accumulated in tumors and controllably released drugs in response to the acidic TME. The therapeutic agents effectively inhibited Hh pathway activation, which consequently suppressed the secretion of extracellular matrix (ECM) components by CAFs, and repolarized M2-like TAMs toward immunostimulatory M1 phenotype. Synergizing with PD-1/PD-L1 inhibition, this strategy significantly enhanced dendritic cells (DCs) maturation and antigen-presenting function, boosted CD8^+^ T cell infiltration and immune response, and effectively suppressed primary tumor progression and metastasis in TNBC murine models. Our findings highlight that the biomimetic nanoplatform based on E-V/B@NM remodels the TME by reprogramming both CAFs and TAMs, thereby overcoming immunosuppression and improving ICB efficacy in TNBC.

## Highlights

• Developed biomimetic nanoplatform (*E*-V/B@NM) for co-delivery of Vismodegib and BMS-1.
• Inhibited Hh pathway to suppress ECM by CAFs and repolarize M2 TAMs to M1 phenotype.
• Synergized with PD-1/PD-L1 blockade to boost CD8^+^ T cells against TNBC.

## Graphical abstract

**Figure:**
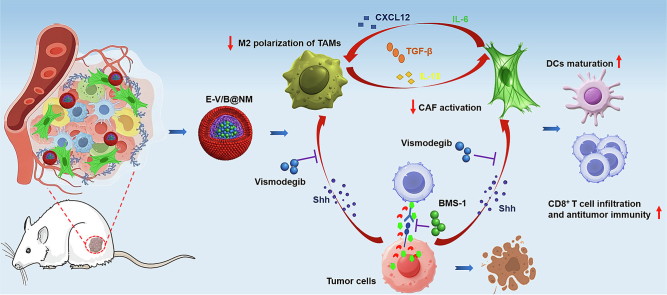

## 1 Introduction

Immunotherapy is a crucial therapeutic approach for triple-negative breast cancer (TNBC). Immune checkpoint blockade (ICB) targeting PD-1/PD-L1 combined with chemotherapy has been approved for advanced or high-risk early-stage TNBC, significantly improving survival outcomes in some patients, particularly those with PD-L1-positive tumors. However, the efficacy of ICB in TNBC remains limited, with objective response rates of only 20%–40% (Dvir et al., 2024). A key factor restricting the effectiveness of ICB in TNBC is the immunosuppressive tumor microenvironment (TME). A subset of TNBC cases is classified as “immune-excluded” subtypes, characterized by minimal cytotoxic T-cell infiltration and poor immune activation. Furthermore, the TME in TNBC is enriched with immunosuppressive cells, such as tumor-associated macrophages (TAMs), which further dampen antitumor immunity and promote immune evasion (Kundu et al., 2024; Xu et al., 2024). Given these challenges, there is an urgent need to explore more optimized combination immunotherapy strategies to enhance therapeutic efficacy in TNBC.

A hallmark feature of the TME in TNBC is the presence of a dense fibrotic stroma, typically characterized by hyperactivated cancer-associated fibroblasts (CAFs) and excessive extracellular matrix (ECM) deposition (Ding et al., 2022). This dense stroma increases solid stress, elevates interstitial fluid pressure, and compresses blood vessels, creating a series of pathological barriers that impede the penetration of cytotoxic T cells into the tumor parenchyma–a phenomenon contributing to tumor immune exclusion (Chen et al., 2019; Xiao et al., 2023). Multiple signaling pathways, including Hedgehog (Hh) signaling, have been implicated in CAF activation and ECM production (Walter et al., 2010; Fang et al., 2023). Tumor cells excessively secrete Hh ligands (*e.g.*, Sonic Hedgehog, Shh), which activate the Hh pathway in adjacent fibroblasts *via* paracrine signaling, driving the differentiation of quiescent fibroblasts into a CAF phenotype. These activated CAFs, in turn, secrete cytokines, growth factors, and ECM-modifying proteins under Hh signaling induction, thereby facilitating tumor immune evasion (Zhang et al., 2021; Giammona et al., 2023). Additionally, Hh ligands secreted by tumor or stromal cells can activate Hh receptors on macrophages in a paracrine or autocrine manner, directly inducing M2-polarization-related gene expression (Petty et al., 2019). The Hh pathway also drives TAMs toward a pro-tumor M2-like phenotype by modulating cytokine secretion and metabolic reprogramming, thereby supporting immune evasion and TME remodeling (Wang et al., 2023). Consequently, Hh pathway inhibition may enhance anti-tumor immunity by suppressing the pro-tumorigenic functions of CAFs and reversing TAM polarization.

**Scheme 1:**
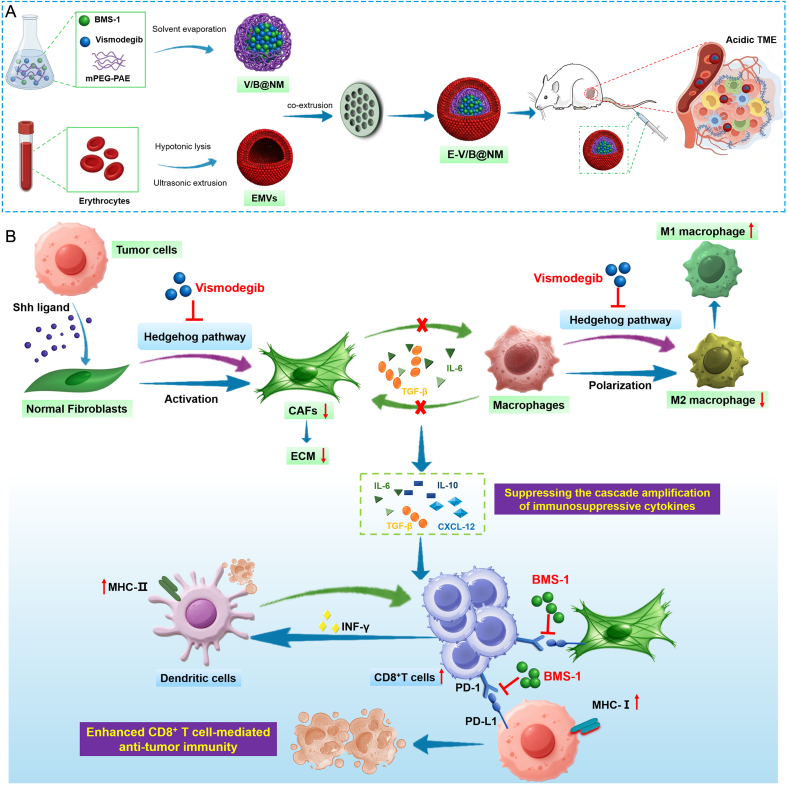
Schematic illustration of the erythrocyte membrane-camouflaged biomimetic co-delivery nanoplatform encapsulating Vismodegib and BMS-1 (E-V/B@NM) for enhancing antitumor immunity in TNBC.

While combination immunotherapy enhances antitumor efficacy, it may concurrently increase the risk of immune-related adverse events and cumulative toxicity. Nanocarrier-based drug delivery platform not only improve the bioavailability of poorly soluble drugs and control release kinetics to prolong therapeutic effects, but also enable precise targeted delivery to reduce off-target toxicity in healthy tissues (Jain et al., 2018; Peng et al., 2023). However, conventional synthetic nanoparticles often suffer from premature clearance by the immune system, leading to suboptimal treatment outcomes. To address this limitation, cell membrane-camouflaged biomimetic delivery nanoparticles have been developed by engineering nanocarriers with natural cell membranes, endowing them with enhanced biocompatibility, immune evasion capability, and prolonged circulation time (Xu et al., 2022; Han et al., 2024; Gu et al., 2021). Importantly, the biomimetic nano-delivery systems based on combined immunotherapy strategies have demonstrated significant improvements in therapeutic efficacy against tumors (Xiong et al., 2021; Chen et al., 2024). Herein, we developed an erythrocyte membrane-camouflaged co-delivery nanoplatform encapsulating Vismodegib and BMS-1 for anti-stromal-immunotherapy against TNBC (as illustrated in Scheme 1). The combination therapy not only attenuates the aberrant activation of CAFs and promotes the repolarization of TAMs toward the anti-tumor M1 phenotype through Hh pathway inhibition, but also synergistically remodels the immunosuppressive TME. This dual approach effectively reverses tumor immune exclusion of cytotoxic CD8^+^ T cells and enhances ICB efficacy for refractory TNBC. Our findings highlight the potential clinical value of the developed nanomedicines as a promising strategy to improve immunotherapy outcomes in TNBC.

## 2 Materials and methods

### 2.1 Materials and reagents

Vismodegib (purity ≥98%) was purchased from Adooq Bioscience (Nanjing, China). BMS-1 (purity >99%) was obtained from MedChemExpress (NJ, USA). The amphiphilic block copolymer methoxy polyethylene glycol-poly (β-amino ester) (mPEG-PAE) (PEG, Mw: 5000 Da; PAE, Mw: 10000 Da), Cy7-PEG_5000_-PAE_10000_, and Cy7 were all sourced from Xi'an Ruixi Biological Technology Co., Ltd. (Xi'an, China). Recombinant murine sonic hedgehog, recombinant murine macrophage colony-stimulating factor (M-CSF), and recombinant murine interleukin-4 (IL-4) were procured from PeproTech (NJ, USA).

### 2.2 Preparation of erythrocyte membrane-camouflaged biomimetic co-delivery nanoplatform encapsulating Vismodegib and BMS-1 (E-V/B@NM)

The V/B@NM was prepared using a modified self-assembly solvent evaporation method. 2 mg Vismodegib (dissolved in DMSO) and 2 mg BMS-1 (dissolved in THF) were mixed with 20 mg of either mPEG-PAE or Cy7-PEG-PAE (dissolved in THF) to form the oil phase, while PBS (pH 7.4) served as the aqueous phase. The oil phase was then gradually introduced into the aqueous phase under ice-bath conditions using ultrasonic emulsification, resulting in a homogeneous milky emulsion. Organic solvents were subsequently removed *via* magnetic stirring under vacuum pressurization conditions. Unincorporated Vismodegib and BMS-1 were eliminated by centrifugation at 4000 rpm for 30 min at 4 °C using Millipore ultrafiltration tubes, yielding the Vismodegib and BMS-1 co-encapsulated nanomicelles (V/B@NM).

Erythrocyte membrane vesicles (EMVs) were prepared through hypotonic swelling and liposome extrusion techniques. Briefly, blood collected from the BALB/c mice was centrifuged to isolate erythrocytes. The cells were subjected to hypotonic swelling in 0.25 × PBS for 2 h, followed by centrifugation at 12,000 rpm for 10 min at 4 °C to obtain erythrocyte membranes. After ultrasonication, the membranes were sequentially extruded through 400 nm, 200 nm, and 100 nm polycarbonate membranes to produce EMVs.

For the final assembly, EMVs and V/B@NM were mixed at a 1:1 mass ratio, homogenized by ultrasonic oscillation, and co-extruded through 400 nm and 200 nm polycarbonate membranes using a liposome extruder. The resulting solution was centrifuged at 1500 rpm for 15 min to remove unincorporated nanomicelles, ultimately yielding the E-V/B@NM. The morphology, particle size distribution, and zeta potential of the fabricated E-V/B@NM were analyzed using transmission electron microscopy (TEM) and dynamic light scattering (DLS) techniques. The encapsulation efficiency (EE) and drug loading capacity (DLC) of both Vismodegib and BMS-1 were quantitatively determined by high-performance liquid chromatography (HPLC). DLC and EE were calculated according to the following definitions and equations. DLC is defined as the percentage of the mass of drug encapsulated in the nanoparticles relative to the total mass of the nanoparticles, reflecting the drug-carrying capacity of the nanocarrier. EE is defined as the percentage of the mass of drug encapsulated in the nanoparticles relative to the total mass of drug initially added during preparation, reflecting the efficiency of drug entrapment. The equations are as follows:

(1) $$\;\mathrm{DLC}\left(\%\right)=\frac{W_{\text{encapsulated}}}{W_{\text{nanoparticles}}}\times 100\%\;\;$$

(2) $$\begin{array}{cccc} & \mathrm{EE}\left(\%\right)=\frac{W_{\text{encapsulated}}}{W_{\text{total}\;\mathrm{fed}}}\times 100\% & & \; \end{array}$$

where $W_{\text{encapsulated}}$ is the mass of drug encapsulated in the nanoparticles, $W_{\text{nanoparticles}}$ is the total mass of the lyophilized nanoparticles, and $W_{\text{total}\;\mathrm{fed}}$ is the total mass of drug initially added during preparation.

### 2.3 pH-triggered drug release of E-V/B@NM

The pH-sensitive drug release profile of E-V/B@NM under different conditions (pH 7.4, pH 6.5, and pH 5.0) was evaluated using the dialysis method *in vitro*. Briefly, three batches of fresh E-V/B@NM suspension were placed in cellulose dialysis bags (8000 Da, Solarbio, Beijing, China). After tightening both ends of the dialysis bags, the test samples were placed in a corked conical flask containing 50 mL of dissolution medium and stirred at 37 °C, 120 rpm. At predetermined time points (0.5, 1, 2, 4, 6, 8, 12, 24, and 48 h), 1 mL of release solution was collected, and an equal volume of fresh dissolution medium was added. Then drug release of E-V/B@NM was analyzed by HPLC, and the cumulative release curves of Vismodegib, and BMS-1 were calculated.

### 2.4 Cell culture, CAF activation, and isolation and polarization of bone marrow-derived macrophages (BMDMs) from mice

Murine embryonic fibroblast cell line (NIH/3T3) and murine breast cancer cell line (4T1) were obtained from Procell Life Science & Technology Co., Ltd. (Wuhan, China). Cells were maintained in DMEM (for NIH/3T3) or RPMI 1640 medium (for 4T1) supplemented with 10% fetal bovine serum and 1% penicillin-streptomycin at 37 °C in a humidified 5% CO_2_ incubator. For CAF activation, confluent NIH/3T3 cells were seeded in 6-well plates and treated with 5 ng/mL Shh ligand for 48 h.

BMDMs were isolated from 6-week-old C57BL/6J mice euthanized by cervical dislocation. The mice were disinfected by immersion in 75% ethanol for 5 min. The hind limbs were then excised at the hip joints, and the femurs and tibias were carefully isolated after removing surrounding muscle tissues. The bone marrow was flushed out using pre-cooled culture medium, and the resulting cell suspension was collected. After red blood cell lysis, the cells were centrifuged at 1500 rpm for 5 min and subsequently cultured in complete DMEM medium supplemented with 25 ng/mL M-CSF for 7 days to obtain mature BMDMs. For M2-like macrophage polarization, the BMDMs were treated with 20 ng/mL IL-4 or Shh for 24 h before further experiments.

### 2.5 Cell immunofluorescence assay

Immunofluorescence assay was conducted following the previously described method (Ma et al., 2025). Briefly, after treatment with different drugs, CAFs were fixed with 4% paraformaldehyde (PFA) for 15 min at room temperature, and then permeabilized with 0.2% Triton X-100 in PBS for 10 min. To reduce non-specific binding, the cells were blocked with 5% BSA in PBS for 1 h at room temperature, followed by incubation with primary antibodies against Smo (1:200, Proteintech), Gli1 (1:200, Proteintech), and α-SMA (1:200, Proteintech) overnight at 4 °C. Thereafter, the cells were incubated with Cy3-conjugated secondary antibody for 1 h at room temperature in the dark. Nuclei were counterstained with DAPI for 5 min. Finally, the cells were visualized using a confocal laser scanning microscope.

### 2.6 Animal models and *in vivo* biodistribution study

Female BALB/c mice (4–6 weeks old, 16 ± 2 g) were purchased from Charles River Laboratories (Beijing, China) and housed in the Laboratory Animal Center of Ningxia Medical University in compliance with institutional animal care guidelines. To establish the orthotopic TNBC model, 4T1 cells (5 × 10^5^ cells/mouse) were injected into the right fourth mammary fat pad of isoflurane-anesthetized mice. All animal experiments were approved by the Institutional Animal Care and Use Committee of Ningxia Medical University (Protocol No. IACUC-NYLAC-2023-156) and conducted in accordance with relevant guidelines.

When the tumor volume in tumor-bearing mice exceeded 500 mm^3^, the mice received intravenous injections of either free Cy7 dye or Cy7-labeled nanocarriers (V/B@NM(Cy7) or E-V/B@NM(Cy7)) at a dose of 100 μL (5 mg/mL). Following administration, the animals were anesthetized and subjected to whole-body imaging at predetermined time points (2, 6, 12, and 24 h) using an IVIS Spectrum imaging system (Caliper PerkinElmer, USA) with excitation/emission wavelengths set at 670/773 nm. Upon completion of the *in vivo* imaging, major organs and tumor tissues were harvested for *ex vivo* imaging analysis. Quantitative analysis of the fluorescence signals was performed using the imaging system's proprietary analysis software.

### 2.7 *In vivo* antitumor efficacy evaluation

On day 9 post-establishment of the orthotopic TNBC mouse model, mice were randomly allocated into four treatment groups (*n* = 6/group): (i) Saline (control); (ii) E-V@NM (biomimetic nanomedicines encapsulating Vismodegib); (iii) E-B@NM (biomimetic nanomedicines encapsulating BMS-1); and (iv) E-V/B@NM. Mice received intravenous injections of either saline or the respective nanomedicines (containing 8 mg/kg Vismodegib and/or 8 mg/kg BMS-1) on days 9, 12, 15, 18, 21, 24, and 30. For the survival study, additional control groups were included: Vismodegib monotherapy; BMS-1 monotherapy; and combination therapy with Vismodegib and BMS-1. The treatment window was extended with additional dosing on days 36, 42, and 48. Tumor volumes were measured every three days using calipers, with volume calculated as: V = π/6 × (L × W^2^), where L = longest diameter and W = shortest diameter. Humane endpoints were applied when tumor volume exceeded 1500 mm^3^. Blood was collected for further complete blood count, and liver and kidney function tests. Tumors and major organs (heart, lung, liver, spleen, and kidney) were harvested and fixed in 4% PFA for subsequent analysis.

### 2.8 Histopathological analysis

The fixed tumor and major organ tissues from mice were sequentially processed through dehydration, paraffin infiltration, and embedding. Subsequently, 5-μm-thick sections were cut and subjected to hematoxylin and eosin (H&E) staining following deparaffinization. For specialized staining, deparaffinized tumor tissue sections were processed according to the manufacturer's protocols of Masson's trichrome staining kit (Beyotime Biotechnology, Shanghai, China), and Enhanced Sirius Red staining kit (Solarbio, Beijing, China). After staining, the sections were dehydrated, mounted, and imaged using light microscopy for subsequent analysis.

### 2.9 Immunohistochemistry (IHC) and multiplex immunohistochemistry (mIHC) assays

For IHC analysis, paraffin-embedded tumor tissue sections were first deparaffinized, followed by antigen retrieval in citrate buffer (pH 6.0) with 15 min of boiling. The sections were then incubated overnight at 4 °C with the following primary antibodies: anti-Ki-67 (1:1000, Proteintech), anti-CD31 (1:4000, Proteintech), anti-PD-L1 (1:500, Proteintech), anti-MHC class I (1:100, Affinity), and anti-MHC class II (1:2000, ABclonal). Subsequently, the sections were treated with HRP-conjugated secondary antibodies from a DAB detection kit. Stained sections were imaged using light microscopy, and protein expression levels were quantified using ImageJ software.

mIHC assay was performed using tyramide signal amplification (TSA) technology. Briefly, after antigen retrieval, tumor tissue sections were incubated with 3% H_2_O_2_ at room temperature. Following blocking, sections were sequentially stained with anti-α-SMA (1:200, Proteintech) and corresponding Cy3-conjugated donkey anti-rabbit IgG (1:500, Servicebio). After washing, sections were incubated with freshly prepared TSA working solution. For subsequent staining rounds, sections underwent repeated antigen retrieval and were incubated with anti-Collagen I (1:1500, Servicebio) and anti-CD8α (1:500, Proteintech) along with their respective fluorophore-conjugated secondary antibodies. Finally, nuclei were counterstained with DAPI, and slides were mounted for imaging using a fluorescence slide scanning system.

### 2.10 Enzyme-linked immunosorbent assay (ELISA)

Cell culture supernatants were collected for analysis. Mouse tumor tissues were homogenized and centrifuged at 3000 rpm for 10 min to obtain the supernatant. The concentrations of Collagen I (Col I), Fibronectin (FN), Hyaluronic acid (HA), TGF-β1, IL-10, IL-12, TNF-α, IL-6, CXCL12, IFN-γ, and CXCL10 were measured using the following commercial ELISA kits according to the manufacturers' protocols of mouse Col I ELISA kit (ml301800, mlbio), mouse FN ELISA kit (ml002131, mlbio), mouse HA ELISA kit (ml500128, mlbio), mouse TGF-β1 ELISA kit (EK00057, ABclonal), mouse IL-10 ELISA kit (MU30055, Bioswamp), mouse IL-12 ELISA kit (RK2183, MULTI SCIENCES), mouse TNF-α high sensitivity ELISA kit (RK04875, ABclonal), mouse IL-6 ELISA kit (EK206, MULTI SCIENCES), mouse CXCL12 ELISA kit (RK00168, ABclonal), mouse IFN-γ ELISA kit (MU30038, Bioswamp), and mouse CXCL10 ELISA kit (RK00056, ABclonal).

### 2.11 Flow cytometry analysis

Flow cytometry was performed according to previously described methods (Zhang et al., 2023a). For *in vitro* experiments, drug-treated cells were trypsinized, centrifuged, and collected for analysis. For *in vivo* experiments, fresh tumor tissues were harvested from mice and processed into single-cell suspensions. The cell suspensions were incubated with the following fluorescently-labeled antibodies (all from BD Biosciences) at 4 °C for 30 min in the dark: PE rat anti-mouse F4/80 (1:100), Alexa Fluor 647 rat anti-mouse CD206 (1:100), PE-Cy7 rat anti-mouse CD86 (1:100), RY610 hamster anti-mouse CD80 (1:100), APC-Cy7 rat anti-mouse CD45 (1:100), FITC rat anti-mouse CD3 (1:100), and PerCP-Cy5.5 rat anti-mouse CD8 (1:100). After staining, cells were washed with Perm/Wash™ buffer and resuspended for flow cytometry analysis.

### 2.12 Statistical analysis

All experiments were performed in triplicate, with data expressed as mean ± SEM. Statistical analyses were conducted using either Origin 9.0 or SPSS 24.0 software. Before selecting the comparative tests, the assumptions of normality and homogeneity of variances were rigorously examined using the Shapiro-Wilk test and Levene's test, respectively. For variables that conformed to a normal distribution and exhibited equal variances, a one-way analysis of variance (ANOVA) was employed to detect significant differences among groups. Conversely, for variables that deviated from a normal distribution or showed unequal variances, the non-parametric Kruskal-Wallis rank-sum test was applied. A *P*-value ≤0.05 was considered statistically significant.

## 3 Results

### 3.1 Characterization of erythrocyte membrane-camouflaged biomimetic co-delivery nanoplatform encapsulating Vismodegib and BMS-1 (E-V/B@NM)

**Fig. 1:**
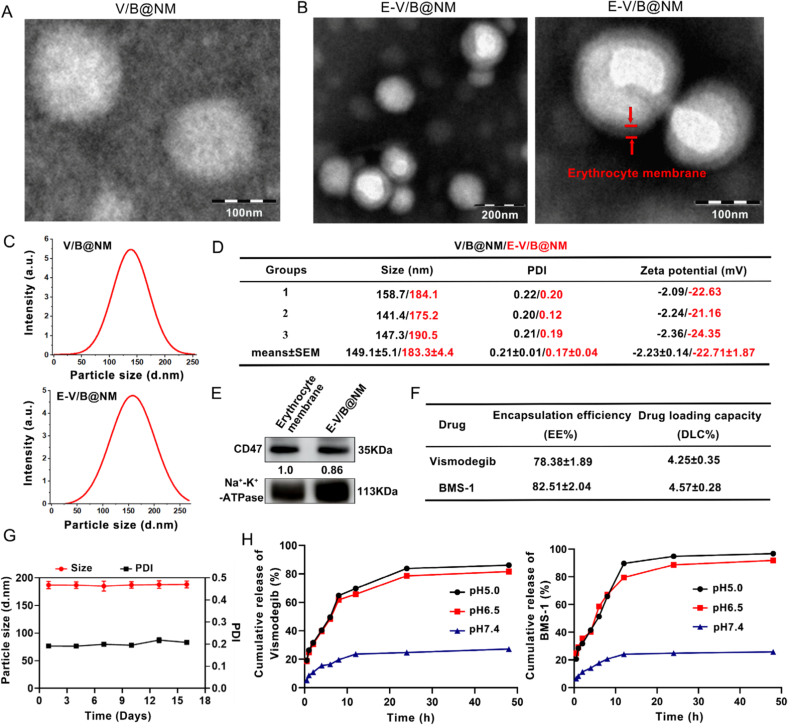
Characterization of the biomimetic nanomedicines E-V/B@NM. (A and B) TEM images of V/B@NM and E-V/B@NM. (C) Size distribution profiles of V/B@NM and E-V/B@NM. (D) Hydrodynamic diameter, PDI, and zeta potential of V/B@NM and E-V/B@NM (n = 3). (E) Expression of erythrocyte membrane marker protein CD47 in E-V/B@NM (n = 3). (F) Encapsulation efficiency and drug loading capacity of Vismodegib and BMS-1 (n = 3). (G) Stability profile of E-V/B@NM over time. (H) Cumulative release kinetics of Vismodegib and BMS-1 under different pH conditions.

The biomimetic nanomedicines co-encapsulating Vismodegib and BMS-1 were successfully fabricated by co-extruding EMVs with V/B@NM. TEM images of Fig. 1A and B revealed that both V/B@NM and E-V/B@NM exhibited spherical morphologies, with measured diameters of 132.5 ± 5.4 nm and 157.2 ± 4.9 nm, respectively. A uniform erythrocyte membrane coating (∼20 nm in thickness) was clearly observed on the surface of E-V/B@NM. Further characterization by DLS demonstrated that the hydrodynamic diameters of V/B@NM and E-V/B@NM were 149.1 ± 5.1 nm and 183.3 ± 4.4 nm, respectively (Fig. 1C and D), with a narrow size distribution and high homogeneity of the prepared nanocarriers. Following erythrocyte membrane coating, the zeta potential of V/B@NM shifted from −2.23 ± 0.14 mV to −22.71 ± 0.14 mV. Additionally, western blot analysis confirmed the presence of the erythrocyte membrane-specific protein CD47 in E-V/B@NM (Fig. 1E), further verifying the successful membrane coating process. Quantitative analysis of the drug loading capacity and encapsulation efficiency of E-V/B@NM by HPLC revealed that the DLC for Vismodegib and BMS-1 were 4.25 ± 0.35% and 4.57 ± 0.28%, respectively, while their EE were 78.38 ± 1.89% and 82.51 ± 2.04%, respectively (Fig. 1F). The particle size and PDI of E-V/B@NM under physiological conditions over time were monitored. Throughout the 17-day observation period, no significant changes in either particle size or PDI were detected, demonstrating the excellent stability of E-V/B@NM (Fig. 1G). To investigate the drug release profile in tumor-mimicking acidic microenvironments, the release kinetics of Vismodegib and BMS-1 from E-V/B@NM under physiological condition (pH 7.4) and mildly acidic conditions (pH 6.5 and 5.0) were examined. As presented in Fig. 1H, both drugs exhibited minimal cumulative release (<23%) at pH 7.4 within 48 h. However, with decreasing pH, the carrier underwent disintegration, leading to progressively enhanced drug release. Notably, a burst release pattern was observed within the first 15 h, culminating in cumulative release rates of 81.6% and 91.1% for Vismodegib and BMS-1, respectively, by 48 h. These results demonstrate the pH-triggered drug release characteristics of E-V/B@NM, highlighting its potential for controlled drug delivery in the acidic TME *in vivo*.

### 3.2 Biomimetic nanomedicines inhibit CAF-mediated ECM component release and reverse M2-like macrophage polarization *in vitro*

**Fig. 2:**
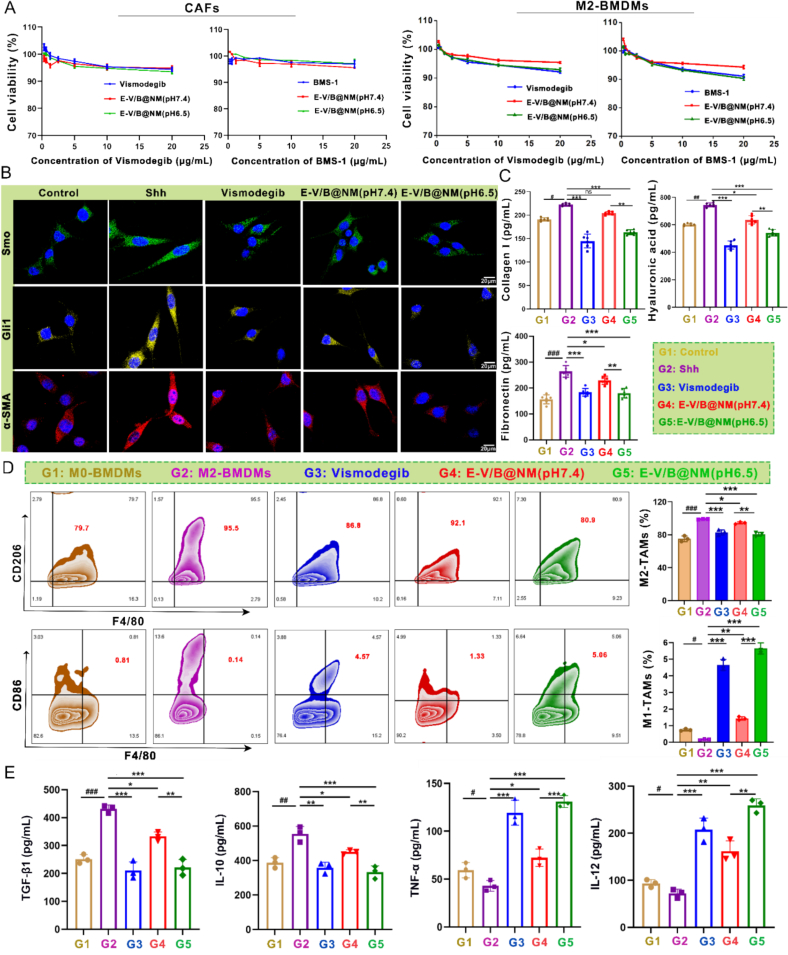
The effect of E-V/B@NM on CAF secretion and TAM polarization *in vitro*. (A) The effect of E-V/B@NM on the viability of Shh-induced NIH/3T3 cells (named CAFs) and M2-BMDMs (n = 6). (B) The effect of E-V/B@NM on protein expression of Smo, Gli1, and α-SMA in CAFs. (C) The effect of E-V/B@NM on secretion of collagen I, fibronectin, and hyaluronic acid by CAFs (n = 6). (D) The effect of E-V/B@NM on polarization of M2-like BMDMs or M1-like BMDMs (n = 3). (E) The effect of E-V/B@NM on secretion of IL-10, TGF-β1, TNF-α and IL-12 by M2-like BMDMs or M1-like BMDMs (n = 3). Data are presented as mean ± SEM. Statistical significance was determined by one-way ANOVA followed by Tukey's *post hoc* test for multiple comparisons. ^#^*P*<0.05, ^##^*P*<0.01, ^###^*P*<0.001; *P < 0.05, **P < 0.01, ***P < 0.001.

To evaluate the cytotoxicity of E-V/B@NM on CAFs and M2-like macrophages, we measured cell viability after 24 h of treatment with E-V/B@NM containing 0.3125–20 μg/mL of Vismodegib or BMS-1. As shown in Fig. 2A, both cell types remained >95% viable at drug concentrations below 5 μg/mL. Between 5 and 20 μg/mL, viability decreased dose-dependently but stayed above 90%. Therefore, 5 μg/mL of either drug in E-V/B@NM was used for subsequent *in vitro* experiments.

Furthermore, we investigated the effect of E-V/B@NM on Hh pathway-related protein expression in CAFs. As shown in Fig. 2B, compared to the control group, Shh-induced NIH/3T3 cells exhibited significantly elevated expression levels of Smo, Gli1 and α-SMA, confirming that 5 ng/mL Shh ligand effectively induces CAF activation through Hh signaling pathway. Upon treatment with E-V/B@NM, the E-V/B@NM(pH 6.5) group demonstrated a marked downregulation of Smo, Gli1 and α-SMA protein levels, compared to the E-V/B@NM(pH 7.4) group. This effect was attributed to the acid-triggered enhanced release of Vismodegib in the acidic condition. Subsequently, we further validated the inhibitory effect of E-V/B@NM on the secretion of ECM components (collagen I, fibronectin, and hyaluronic acid) by CAFs using ELISA. As shown in Fig. 2C, E-V/B@NM significantly suppressed the Shh-induced overproduction of these ECM proteins under acidic conditions. Moreover, incubation of M0-BMDMs with 20 ng/mL Shh for 24 h induced macrophage polarization toward the M2 phenotype, increasing the proportion of F4/80^+^CD206^+^ macrophages from 75.1 ± 2.0% to 98.9 ± 0.2% (Fig. 2D). In contrast, treatment with Vismodegib significantly reduced the M2-like macrophage population. Administration of E-V/B@NM under weakly acidic conditions (pH 6.5) markedly reversed M2 polarization compared to physiological conditions (pH 7.4), decreasing the M2-like macrophage proportion to 80.0 ± 1.0%. Importantly, both Vismodegib and E-V/B@NM(pH 6.5) increased F4/80^+^CD86^+^ macrophages from 0.17 ± 0.02% to 4.7 ± 0.2% and 5.7 ± 0.2%, respectively. ELISA results (Fig. 2E) confirmed that E-V/B@NM(pH 6.5) reversed M2 polarization, significantly downregulating the expression of M2 macrophage functional markers (TGF-β1 and IL-10) while upregulating M1 functional markers (TNF-α and IL-12) compared to M2-BMDMs. These findings collectively demonstrate that E-V/B@NM effectively suppresses CAF activation, diminishes their ECM-secreting capacity, and promotes macrophage repolarization from the M2 to the M1 phenotype by antagonizing the Hh signaling pathway.

### 3.3 E-V/B@NM enhances tumor-targeted distribution in orthotopic TNBC mouse models

**Fig. 3:**
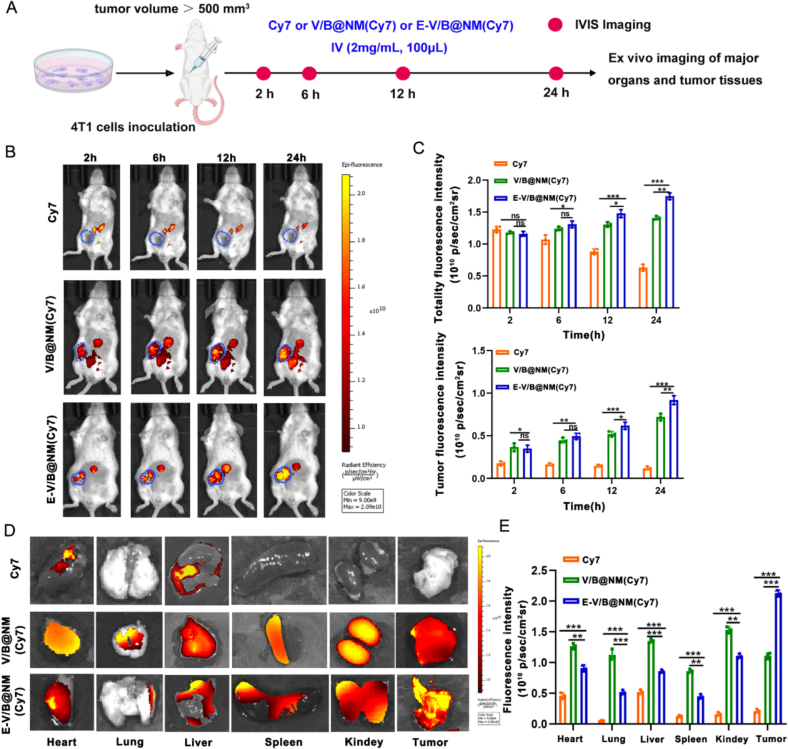
Distribution and *in vivo* imaging of E-V/B@NM(Cy7) in 4T1 orthotopic TNBC mice. (A) Schematic diagram of biodistribution assessment for E-V/B@NM(Cy7). (B and C) Distribution, totality fluorescence intensity and tumor fluorescence intensity of Cy7, V/B@NM(Cy7) and E-V/B@NM(Cy7) at various times in 4T1 orthotopic TNBC mice (n = 3). (D and E) *Ex vivo* imaging and fluorescence intensity of Cy7, V/B@NM(Cy7) and E-V/B@NM(Cy7) in isolated major organs and tumors (n = 3). Data are presented as mean ± SEM. Statistical significance was determined by one-way ANOVA followed by Tukey's *post hoc* test for multiple comparisons. *P < 0.05, **P < 0.01, ***P < 0.001, “ns” indicates no statistically significant difference.

The ability of nanomedicines to target tumors is key to their antitumor effect. IVIS imaging was used to track the distribution of E-V/B@NM (see timeline in Fig. 3A). As shown in Fig. 3B and C, free Cy7 dye showed no increase in tumor fluorescence over time, because small molecules like Cy7 are quickly cleared by diffusion. In contrast, the nanocarriers selectively accumulated in solid tumors *via* the enhanced permeability and retention effect. Compared with V/B@NM, E-V/B@NM exhibited significantly enhanced accumulation in tumor-bearing mice, with a pronounced preference for tumor that became more evident over time. *Ex vivo* imaging analysis of tumors and major organs revealed substantially higher Cy7 fluorescence intensity in heart, liver, spleen, and kidney tissues from the V/B@NM group. Conversely, the E-V/B@NM group showed stronger fluorescence in tumor tissue along with markedly reduced signals in other organs (Fig. 3D and E). These findings demonstrate that E-V/B@NM, by inheriting the surface proteins of the erythrocytes, exhibits enhanced biocompatibility, reduced immune clearance, and improved tumor-targeted delivery efficiency.

### 3.4 E-V/B@NM-based combination immunotherapy enhances therapeutic efficacy with biosafety in TNBC mice

**Fig. 4:**
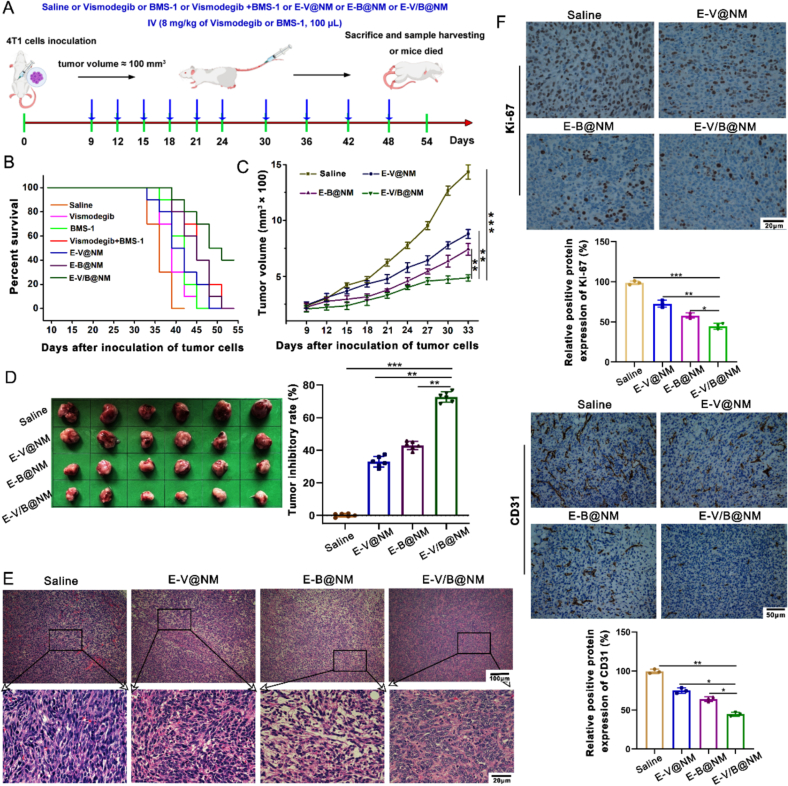
*In vivo* antitumor efficacy of the biomimetic co-delivery nanoplatform based combination therapy. (A) Establishment of the syngeneic tumor model and a schematic diagram of drug treatment. (B) Survival time monitoring of tumor-bearing mice after treatment. (C) Tumor volume in different treatment groups (n = 6). (D) Representative tumor images and tumor inhibitory rate in different treatment groups (n = 6). (E) Histopathological analysis of tumors in different treatment groups. (F) Immunohistochemical analysis of Ki-67 and CD31 of tumors in different treatment groups (n = 3). Data are presented as mean ± SEM. Statistical significance was determined by one-way ANOVA followed by Tukey's *post hoc* test for multiple comparisons. *P < 0.05, **P < 0.01, ***P < 0.001.

We first assessed how E-V/B@NM affects the survival of tumor-bearing mice (administration schedule shown in Fig. 4A). At day 45, survival rates ranged from 0% (saline, Vismodegib, BMS-1) to 70% (E-V/B@NM), and by day 54, only the E-V/B@NM group maintained 40% survival, demonstrating that E-V/B@NM significantly prolongs the survival time of tumor-bearing mice (Fig. 4B). Building upon the established premise that biomimetic nanomedicines enhance antitumor efficacy compared to free drugs, this study further evaluated the inhibitory effects of nanomedicines on tumor progression. As presented in Fig. 4C, tumor volume exhibited exponential growth in the saline group, while treatment with either E-V@NM or E-B@NM monotherapy decelerated tumor growth. Notably, the combination therapy (E-V/B@NM) of Vismodegib and BMS-1 demonstrated superior therapeutic efficacy, significantly suppressing tumor growth and causing tumor stasis in later stages. Fig. 4D provides quantitative support, showing that E-V/B@NM achieved a tumor inhibition rate of 71.4 ± 4.3% in TNBC xenografts, which was significantly higher than that of E-V@NM (36.4 ± 3.6%) or E-B@NM (43.8 ± 2.2%). Histopathological analysis showed malignant features (*e.g.*, nuclear enlargement, mitotic figures) in saline group, whereas all treatments induced cell shrinkage, vacuolization, and chromatin margination–most pronounced with E-V/B@NM (Fig. 4E). IHC assessment of Ki-67 and CD31 expression demonstrated that E-V/B@NM treatment most effectively suppressed nuclear proliferation capacity and significantly reduced microvessel density, indicating potent inhibition of both tumor cell proliferation and angiogenesis (Fig. 4F).

**Fig. 5:**
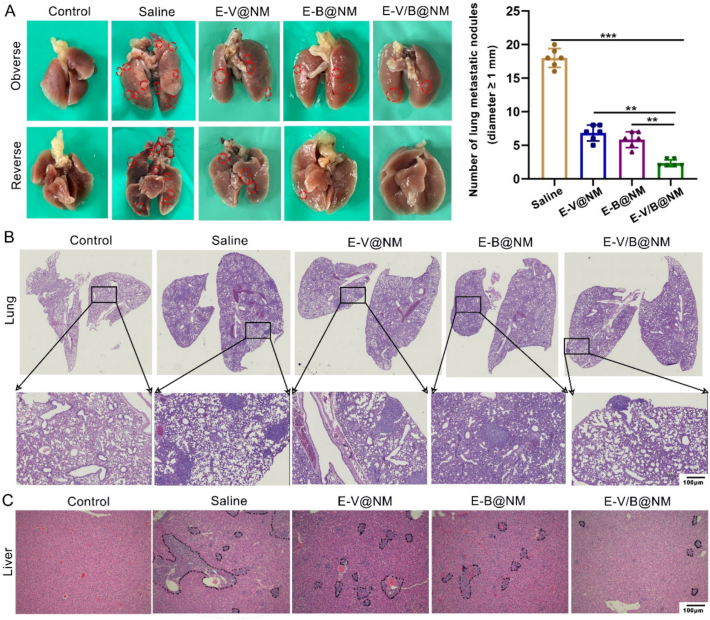
The biomimetic co-delivery nanoplatform based combination therapy inhibits the lung and liver metastasis of tumor cells in 4T1 orthotopic TNBC mice. (A) Representative photographs of tumor metastasis in lungs of mice and statistical analysis of lung metastasis nodules in mice (n = 6). (B and C) Histopathological analysis of tumor metastasis in lung tissues and liver tissues after the biomimetic nanomedicines encapsulating Vismodegib and BMS-1 treatment in orthotopic TNBC mice. Data are presented as mean ± SEM. Statistical significance was determined by one-way ANOVA followed by Tukey's *post hoc* test for multiple comparisons. **P < 0.01, ***P < 0.001.

Metastatic burden analysis showed the saline group developed 17.3 ± 3.2 pulmonary metastatic nodules (diameter ≥ 1 mm), while E-V@NM, E-B@NM, and E-V/B@NM treatments reduced this to 6.8 ± 2.2, 5.8 ± 2.3, and 2.4 ± 1.6 nodules, respectively (Fig. 5A). Subsequent H&E staining of lung and liver tissues revealed extensive metastatic lesions in model animals, whereas all treatments reduced both the number and size of metastatic foci, with E-V/B@NM exhibiting the most pronounced antimetastatic effects (Fig. 5B and C). These results demonstrate that biomimetic nanomedicines co-encapsulating Vismodegib and BMS-1 suppress primary tumor growth and lung/liver metastasis more effectively than single-agent nanosystems.

**Fig. 6:**
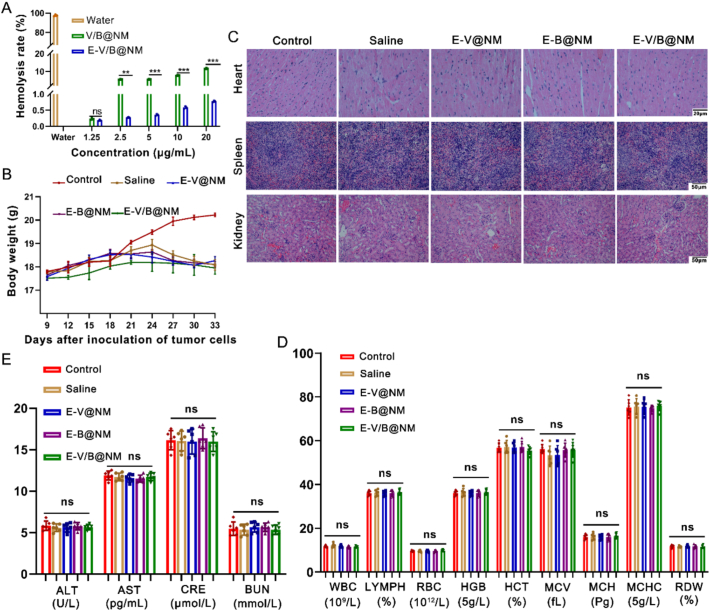
Biosafety evaluation of the biomimetic co-delivery nanoplatform based combination therapy. (A) Hemocompatibility assessment of V/B@NM and E-V/B@NM (n = 3). (B) Body weight changes in mice across different treatment groups (n = 6). (C) Histopathological analysis of major organs (heart, spleen, and kidney) in mice from different treatment groups. (D) Serum levels of hepatic and renal function biomarkers in mice from different treatment groups (n = 6). (E) Hematological parameter changes in mice from different treatment groups (n = 6). Data are presented as mean ± SEM. Statistical significance was determined by one-way ANOVA followed by Tukey's *post hoc* test for multiple comparisons. **P < 0.01, ***P < 0.001, “ns” indicates no statistically significant difference.

Furthermore, the biosafety of nanomedicines was evaluated. As shown in Fig. 6A, compared with V/B@NM, E-V/B@NM demonstrated significantly reduced hemolysis rates, indicating that the biomimetic modification with erythrocyte membranes endowed E-V/B@NM with excellent blood compatibility. Mice treated with E-V@NM or E-B@NM showed early mild weight gain but late decline linked to tumor burden, whereas the E-V/B@NM group exhibited sustained early gain followed by stable late weight (Fig. 6B). Further histopathological analysis of the heart, spleen, and kidney tissues revealed no significant pathological damage in mice after multiple intravenous administrations of E-V/B@NM (Fig. 6C). This observation can be attributed to the erythrocyte membrane coating, which reduces the nonspecific accumulation of the nanocarriers in off-target organs, further validating the results presented in Fig. 3. Additionally, Complete blood count analysis (Fig. 6D) showed no significant differences in key hematological parameters (WBC, LYMPH, RBC, HGB, HCT, MCV, MCH, MCHC, RDW) between treatment groups and the saline group. Similarly, liver function biomarkers (ALT, AST) and kidney function indicators (CRE, BUN) showed no significant changes in any treatment group compared with the saline group (Fig. 6E). These results indicate that the biomimetic nanomedicines have excellent *in vivo* biosafety.

### 3.5 E-V/B@NM-based combination immunotherapy overcomes CAF- and TAM-mediated immunosuppression to enhance CD8^+^ T cell infiltration and antitumor immunity

**Fig. 7:**
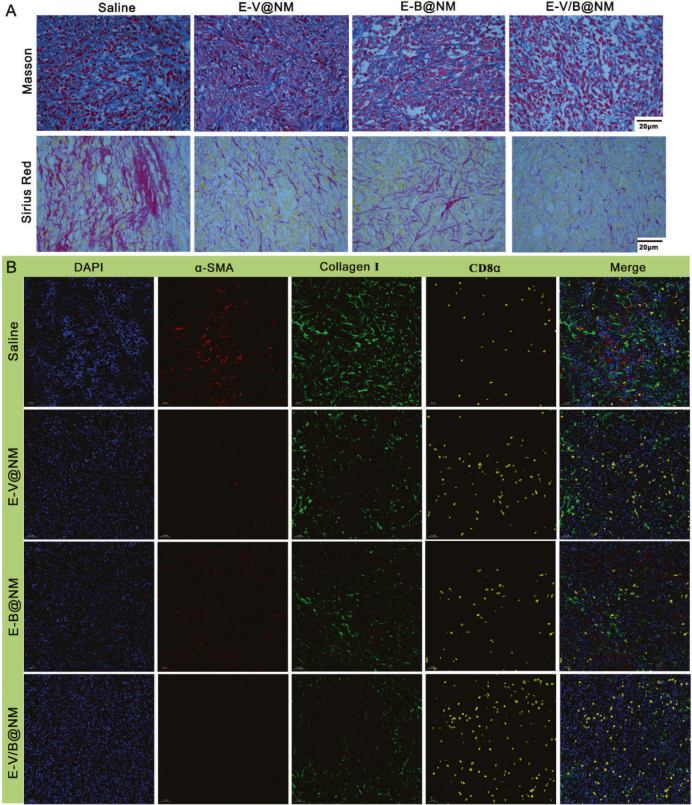
The biomimetic co-delivery nanoplatform based combination therapy-mediated remodeling of ECM potentiates intratumoral infiltration of CD8^+^ T cells. (A) Collagen fiber deposition in the tumor stroma in different treatment groups by Masson staining and Sirius red staining. (B) The expression of α-SMA, collagen I, and CD8α in tumor tissues across treatment groups by mIHC analysis. (For interpretation of the references to colour in this figure legend, the reader is referred to the web version of this article.)

**Fig. 8:**
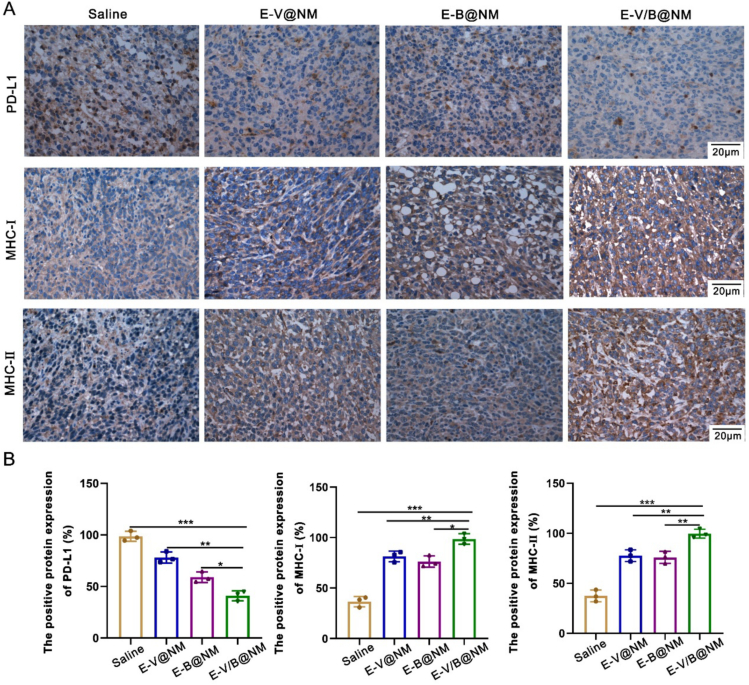
The biomimetic co-delivery nanoplatform based combination therapy enhances tumor immunogenicity. (A and B) Representative images and statistical analysis of PD-L1, MHC-I, and MHC-II expression in tumor tissues in different treatment groups (n = 3). Data are presented as mean ± SEM. Statistical significance was determined by one-way ANOVA followed by Tukey's *post hoc* test for multiple comparisons. *P < 0.05, **P < 0.01, ***P < 0.001.

To further elucidate the mechanism through which E-V/B@NM improves the therapeutic outcomes of ICB therapy, we first evaluated the effect of E-V/B@NM on collagen fiber content in tumor. As shown in Fig. 7A, the saline group exhibited high collagen fiber content, indicating significant ECM fibrosis. Treatment with E-V@NM, E-B@NM, and E-V/B@NM differentially reduced tumor collagen content, with E-V/B@NM showing the most pronounced reduction in both blue-stained (Masson's) and red-stained (Sirius red) collagen fibers. Further analysis of activated CAF biomarker α-SMA, ECM component collagen I, and cytotoxic T cell marker CD8α in tumor tissues revealed that the saline group exhibited high expression of α-SMA and collagen I but low CD8α expression (Fig. 7B). These findings suggest that CAF-derived ECM components create a dense stromal barrier that limits CD8^+^ T cell infiltration, resulting in immune exclusion. Following combination therapy with E-V/B@NM for co-delivery of Vismodegib and BMS-1, the blockade of the Hh pathway disrupted tumor cell-CAF crosstalk. This intervention suppressed α-SMA^+^ CAF activation, resulting in decreased expression of collagen I in the tumor stroma. Furthermore, the combined treatment with BMS-1 downregulated PD-L1 expression in both tumor cells and stroma cells (*e.g.*, CAFs and TAMs), as evidenced by the data presented in Fig. 8A, thereby reversing their collaboratively established immunosuppressive TME to promote intratumoral infiltration of CD8^+^ T cells. Notably, significant upregulation of MHC-I/II expression was also observed in tumors treated with either E-V@NM or E-B@NM monotherapy, with the most pronounced effect seen in the E-V/B@NM combination group (Fig. 8A and B). The results demonstrate that E-V/B@NM effectively reprograms the TME from an immunosuppressive state to an immunostimulatory one, thereby enhancing the capacity of the immune system to recognize tumor cells.

**Fig. 9:**
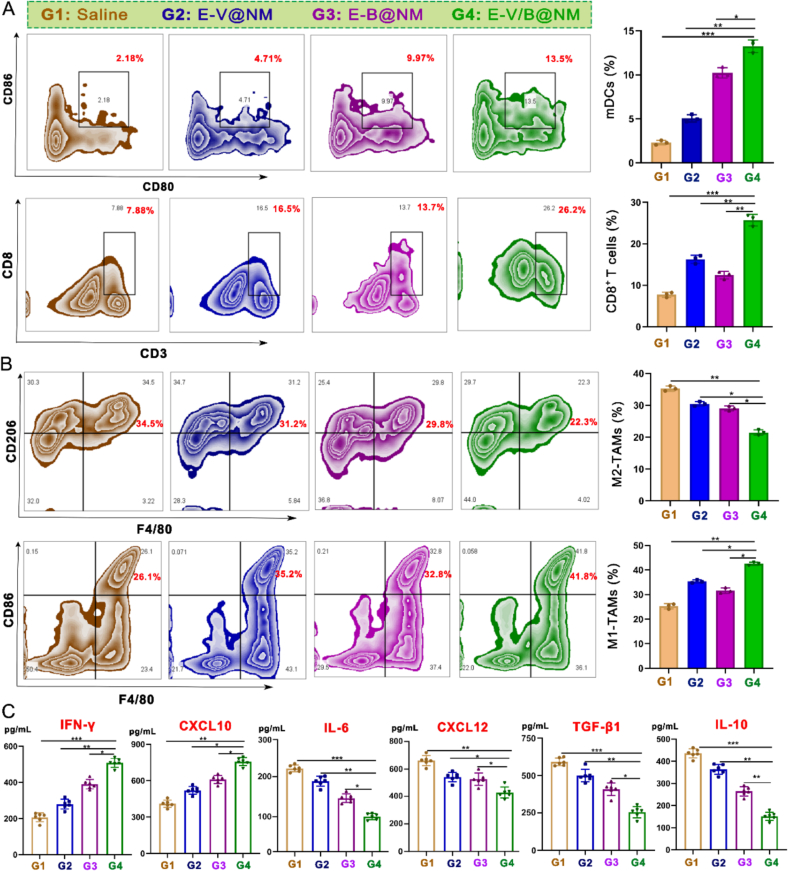
The biomimetic co-delivery nanoplatform based combination therapy decreases the proportions of M2-like TAMs to enhance intratumoral T-cell infiltration and effector T-cell function. (A) Representative cytometric dot plots of mDCs and CD3^+^CD8^+^ T cells in tumors (left) and the proportions of mDCs and CD8^+^ T cells in tumors in different treatment groups (right) (n = 3). (B) Representative cytometric dot plots of F4/80^+^CD86^+^ M1-TAMs cells and F4/80^+^CD206^+^ M2-TAMs cells in tumors (left) and the proportions of M1-TAMs and M2-TAMs in tumors in different treatment groups (right) (n = 3). (C) The protein content of INF-γ, CXCL10, IL-6, CXCL12, TGF-β1, and IL-10 in tumors by ELISA (n = 3). Data are presented as mean ± SEM. Statistical significance was determined by one-way ANOVA followed by Tukey's *post hoc* test for multiple comparisons. *P < 0.05, **P < 0.01, ***P < 0.001.

Flow cytometry further showed that the combination therapy E-V/B@NM significantly increased the infiltration of mature dendritic cells (mDCs) and CD8^+^ T cells in mouse tumors. As shown in Fig. 9A, compared to the saline group and single-agent groups (E-V@NM or E-B@NM), E-V/B@NM markedly enhanced mDC infiltration. CD8^+^ T cell infiltration increased by approximately 18.3%, 9.7%, and 12.5%, respectively, indicating effective priming of an anti-tumor T cell response. Given that TAMs contribute to immunosuppression in TNBC, we further investigated the regulatory impact of E-V/B@NM on TAMs *in vivo*. Fig. 9B demonstrates that E-V/B@NM treatment significantly reduced M2-TAMs infiltration (by approximately 12.2%) compared to the saline group, and also showed superior efficacy over single-agent treatments. Conversely, the combination therapy markedly drives the M1-polarization of TAMs (increased by ∼15.7% *versus* the saline group), consistent with the findings in Fig. 2D. Furthermore, analysis of immunomodulatory cytokines revealed that E-V/B@NM co-delivering Vismodegib and BMS-1 significantly downregulated immunosuppressive cytokines (IL-6, CXCL12, TGF-β1, and IL-10) while upregulating immunostimulatory cytokines (CXCL10 and IFN-γ) in tumors (Fig. 9C). This provides further evidence supporting the transition of the TME toward an immunologically activated state. These findings demonstrate that E-V/B@NM effectively disrupts the tumor cell-CAF-TAM interaction through dual blockade of the Hh signaling pathway and PD-1/PD-L1 axis, which potentiates CD8^+^ T-mediated antitumor immune responses, thereby improving immunotherapeutic efficacy against TNBC.

## 4 Discussion

Immunotherapy utilizing immune checkpoint inhibitors represents a pivotal approach in the treatment of TNBC; however, its efficacy is often limited by the immunosuppressive TME. CAFs and TAMs, as the predominant immunosuppressive cell populations within the TME, engage in complex intercellular crosstalk that establishes a positive feedback loop, collectively suppressing anti-tumor immunity (Mao et al., 2021). Therefore, combining anti-PD-1/PD-L1 therapy with strategies aimed at reprogramming CAFs and TAMs may enhance T cell infiltration into tumors and restore their antitumor functions.

Tumor cell-derived Shh ligand aberrantly activates the Hh signaling pathway in adjacent fibroblasts, promoting their transformation into CAFs and enhancing their ECM remodeling capacity (Walter et al., 2010). Accumulated evidence indicates that the Hh pathway drives TAMs toward M2 polarization through both direct activation of Gli-dependent transcription in macrophages and indirect remodeling of the TME. However, Hedgehog pathway inhibitors (*e.g.*, Vismodegib) can reverse this M2 polarization (Cascio et al., 2021). In this study, we successfully developed an acid-responsive erythrocyte membrane**-**camouflaged nanomedicine for the co-delivery of Vismodegib and BMS-1. The erythrocyte membrane coating serves as a novel nanocarrier strategy, in which the CD47-SIRPα interaction effectively inhibits immune clearance and significantly prolongs the systemic circulation time of the nanoparticles. Its low immunogenicity reduces accumulation in the liver and spleen, and improves tumor-targeted accumulation and drug release (Xia et al., 2019; Tang et al., 2024). The *in vivo* distribution studies further confirm that this biomimetic nanosystem significantly increases drug accumulation in tumor tissues while reducing retention in non-target organs.

The excessive deposition of ECM components such as collagen fibers in tumor stroma creates a fibrotic TME. This fibrotic niche establishes a profoundly immunosuppressive milieu through multidimensional mechanisms, including physical barrier formation, secretion of immunomodulatory molecules (*e.g.*, TGF-β, CCL2, IL-6, CXCL12) that recruit immunosuppressive cells (Fang et al., 2023; Du et al., 2023; Arpinati et al., 2024). CAFs, particularly myofibroblastic CAFs (myCAFs), serve as the primary architects of tumor ECM. These activated fibroblasts secrete various ECM components including collagen, fibronectin, and hyaluronic acid, thereby increasing ECM stiffness, structural stability, and fibrosis (Croizer et al., 2024). This study demonstrates a negative correlation between α-SMA and CD8α expression in tumor tissues from the saline-treated group, suggesting that the dense stromal structure formed by myCAFs inhibits CD8^+^ T cell infiltration. In contrast, treatment with *E*-V/B@NM remodeled the ECM, resulting in a looser architecture and promoting the infiltration of CD8^+^ T cells. Although the study by Nina G. Steele et al (Steele et al., 2021). on pancreatic cancer demonstrated that Hh pathway is uniquely activated in fibroblasts and differentially elevated in myCAFs compared to inflammatory CAFs (iCAFs), with Hh pathway inhibition reducing myCAF abundance while increasing iCAF number–a shift correlated with decreased cytotoxic T cell infiltration–our findings appear contradictory to these observations. This discrepancy may be attributed to two key factors: (1) differences in tumor context and treatment duration, and (2) potential synergistic effects of Vismodegib and BMS-1 combination therapy on remodeling the immunosuppressive TME.

This study further confirms that the E-V/B@NM combination therapy can effectively reprogram polarized M2-type TAMs into an M1-like phenotype. The underlying mechanism primarily involves two aspects: Vismodegib inhibits M2-like macrophage polarization by antagonizing the Hh signaling pathway (Hinshaw et al., 2021), while BMS-1 directly suppresses M2 polarization by blocking the PD-1 signaling in macrophages (Zhang et al., 2023b; Kumar et al., 2024). Additionally, BMS-1 may facilitate M1 polarization indirectly by restoring T cell function and promoting IFN-γ secretion (Li et al., 2024). Importantly, the E-V/B@NM combination therapy effectively disrupts the positive feedback loop between CAFs and TAMs by suppressing the cascade amplification of key immunosuppressive cytokines, such as IL-6, CXCL12, TGF-β1, and IL-10.

Moreover, E-V/B@NM downregulates PD-L1 expression in both tumor cells and stromal cells, such as CAFs and TAMs, thereby reversing the immunosuppressive TME created by these cells. This dual mechanism synergistically promoted intratumoral infiltration of CD8^+^ T cells, as evidenced by Amy J Petty’ study (Petty et al., 2021). Furthermore, the E-V/B@NM combination therapy upregulates the expression of MHC-I/II in tumor tissues. Beyond the established mechanism whereby Hh pathway inhibition reduces secretion of immunosuppressive cytokines by activated CAFs, thereby alleviating their suppressive effect on tumor MHC expression (Otsuka et al., 2015), emerging evidence suggests that Hh inhibition enhances antigen presentation by DCs, indirectly promoting MHC-II expression (Giammona et al., 2024). More importantly, PD-1/PD-L1 blockade leads to activation of CD8^+^ T, which secrete IFN-γ that can directly induce upregulation of MHC-I/II in both tumor cells and antigen-presenting cells (Rodig et al., 2018; Cui et al., 2025). These findings indicate that, compared to E-B@NM monotherapy, the E-V/B@NM combination therapy induces a stronger therapeutic response in refractory TNBC through successful activation of CD8^+^ T cells within the TME, thereby initiating a self-sustaining and progressively enhanced anti-tumor immune cycle.

## 5 Conclusion

In conclusion, the study successfully developed an erythrocyte membrane-camouflaged biomimetic nanomedicine (E-V/B@NM), which demonstrated pH-responsive drug release, excellent biocompatibility, tumor-targeting capability, and biosafety. *In vitro* experiments revealed that E-V/B@NM-mediated Hh pathway inhibition not only suppressed CAF activation and reduced the secretion of ECM components, but also promoted the repolarization of TAMs from the pro-tumoral M2 phenotype to the antitumoral M1 phenotype. *In vivo* studies demonstrated that E-V/B@NM combination therapy significantly inhibited the growth of orthotopic tumors and the formation of pulmonary/hepatic metastatic foci in mice, exhibiting superior efficacy compared to single-agent treatments (E-V@NM or E-B@NM). The tumor growth inhibition rate and metastasis suppression rate reached approximately 71.4% and 88.2%, respectively. The enhanced therapeutic efficacy can be attributed to the synergistic blockade of stroma-tumor interactions by the Vismodegib/BMS-1 combination, which overcomes CAF- and TAM-mediated immunosuppression, reprograms the immunosuppressive TME, promotes DCs maturation and antigen-presenting function, thereby augmenting CD8^+^ T cell tumor infiltration and antitumor immune responses. This study provides a novel therapeutic strategy for the clinical management of TNBC.

## CRediT authorship contribution statement

**Haiwang Luo:** Writing – original draft, Investigation, Data curation, Conceptualization. **Di Cao:** Investigation, Data curation, Conceptualization. **Mei Ma:** Investigation, Formal analysis, Data curation. **Xinyi Lu:** Validation, Methodology. **Jing Wang:** Writing – original draft, Supervision, Project administration.

## Funding

This work was supported by the 10.13039/501100001809National Natural Science Foundation of China (NSFC Grant No. 82503815) and the Central Government-Guided Local Sci-Tech Development Project of Ningxia (Grant No. 2024FRD05104).

## Declaration of competing interest

The authors declare that they have no known competing financial interests or personal relationships that could have appeared to influence the work reported in this paper.

## Data Availability

All data are available from the corresponding authors on reasonable request.
